# Knockdown of mitochondrial sirtuin *sir-2.2* reduces alpha-synuclein clearance and impairs energy homeostasis in a model of ageing

**DOI:** 10.1242/dmm.052197

**Published:** 2025-11-10

**Authors:** Anam Naseer, Pranoy Toppo, Mahmood Akbar, Aamir Nazir

**Affiliations:** ^1^ Academy of Scientific and Innovative Research (AcSIR), Ghaziabad 201002, India; ^2^ Division of Neuroscience and Ageing Biology, CSIR-Central Drug Research Institute, Lucknow 226031, India

**Keywords:** *sir-2.2*, Mitochondria, Neurodegeneration, Parkinson's disease, *Caenorhabditis elegans*

## Abstract

Mitochondria are the regulators of energy production and play a vital role in modulating ageing and age-associated diseases. We investigated the role of sirtuins, a well-studied class of longevity-associated proteins (NAD^+^-dependent histone deacetylases), in mitochondrial biology and Parkinson's disease pathology. In particular, we endeavoured to study the functional implications of the mitochondrial sirtuin *sir-2.2* (orthologue of human *SIRT4*) in regulating neuroprotection in a *Caenorhabditis elegans* model of ageing. We observed that, upon *sir-2.2* knockdown, alpha-synuclein aggregation was increased and expression of the dopamine transporter *dat-1* was reduced. Also, the levels of markers of innate immunity, oxidative stress, mitophagy, mitochondrial unfolded protein response and autophagy were decreased, suggesting an important function of *sir-2.2* in maintaining mitochondrial homeostasis, regulating protein clearance and ameliorating the disease condition. Because of their crucial role in regulating oxidative stress and mitochondrial quality control, studying mitochondrial sirtuins will provide therapeutic insights into the metabolic regulation of ageing and neurodegeneration.

## Teaser

**Summary:** Knockdown of the mitochondrial sirtuin *sir-2.2* aggravates Parkinson's disease pathology and disrupts mitochondrial biology in *Caenorhabditis elegans.*

## INTRODUCTION

Neurodegeneration is an age-associated phenomenon, which detrimentally affects a person's ability to perform routine tasks. It involves the aggregation of various proteins either due to lack of protein clearance or due to improper protein folding (Barbuti et al., 2024 preprint). Although a plethora of work has been done in understanding neurodegeneration, an absolute cure has not yet been found. In addition to rehabilitation and gene therapies, drugs such as levodopa, donepezil, carbidopa, baclofen and apomorphine (Mathur et al., 2023), to name a few, are known to manage the disease symptoms, but they fail to completely heal the condition. The most prominent form of neuromotor disorder that affects millions of people across the globe is Parkinson's disease (PD). According to a World Health Organization (WHO) fact sheet (WHO, 2023), the rate of PD has increased twofold in the past 25 years. In 2019, it was estimated that more than 8.5 million people had PD globally (WHO, 2023)

PD is a late-occurring progressive disorder marked by clustering of alpha-synuclein protein in the substantia nigra pars compacta region of the brain, forming Lewy bodies in the synaptic terminals and blocking the flow of information across the neurons via neurotransmitters such as dopamine. The alpha-synucleopathy results in involuntary shaking and tremors, rigidity, bradykinesia, postural disturbances and dementia (Morris et al., 2024). It occurs owing to complex interactions among multiple predisposing genes [*SNCA*, *LRRK2*, *PRKN*, *DJ1* (also known as *PARK7*), *PINK1* and *ATP13A2*] (Morris et al., 2024), environmental factors (such as pesticides and metal ions), mitochondrial dysfunction, impairment of the protein degradation pathway and oxidative damage (Nuytemans et al., 2010). The physiology and metabolism of an organism play a pivotal role in regulating the progression of the disease.

PD is age associated and thus represents one of the potential consequences of ageing. One of the important ageing hallmarks associated with neurodegeneration is mitochondrial disruption (Hung et al., 2010; Lezi and Swerdlow, 2012; Cheng et al., 2016; Hou et al., 2019; López-Otín et al., 2023; Wodrich et al., 2024). Besides being the powerhouse of the cell and generating energy in the form of ATP, mitochondria are also an integral part of the biosynthesis pathway of different biomolecules such as lipids, iron, amino acids and nucleotides. They also plays a vital role in modulating energy homeostasis, redox balance, stress response pathways and lifespan (Fang et al., 2016; Jain and Zoncu, 2022; Calabrese et al., 2024; Di Noia et al., 2024; Moisoi, 2024). Sirtuins represent an important class of age-associated proteins (NAD^+^-dependent deacetylases), widely studied for their role in longevity (Satoh et al., 2017; Lee et al., 2019). They are evolutionarily conserved in nature and are involved in the regulation of many biological processes, such as cellular senescence, transcription regulation, DNA repair, neuroprotection, mitochondrial metabolism, genome stability, etc. (Naseer et al., 2021). Among sirtuins, those that regulate mitochondrial homeostasis are known as mitochondrial sirtuins (Wątroba and Szukiewicz, 2016; Gupta et al., 2022; Xiao et al., 2023). These regulate components of multiple signalling processes, such as AMPK, NF-κB, p53 and mTOR (Lautrup et al., 2019; Xiao et al., 2023), and are involved in balancing the electron transport system and ATP production (Dhiman et al., 2024; Kim et al., 2024).

Although mitochondrial sirtuins are an integral part of various metabolic processes, their role in regulating lifespan and neurodegeneration has not been widely explored. Thus, in this study, we focused on investigating the role of mitochondrial sirtuins in regulating longevity and neurodegeneration in an exceptional model of ageing, *Caenorhabditis elegans*. In *C. elegans*, mitochondrial sirtuins are encoded by two genes: *sir-2.2* and *sir-2.3* (Viswanathan and Tissenbaum, 2013). *C. elegans* is a tiny nematode of 1 mm in length, but offers great potential as an animal model as it has ∼70% genetic homology with human, a fixed number of cells (302 neurons), whole-genome mapping, cellular complexity and conservation of disease pathways (Harris, 2004; Corsi et al., 2015). Both *C. elegans* mitochondrial sirtuins show functional overlap but exert their effects via different mechanisms. Comparatively, of the two genes, our initial screening showed more prominent effects of *sir-2.2* gene knockdown (KD) on reducing mitochondrial content (Fig. S1); thus, here, we investigated *sir-2.2*-mediated mitotherapy in regulating ageing and neurodegeneration.

## RESULTS

### *sir-2.2* KD increases alpha-synuclein aggregation and detrimentally affects dopaminergic neuronal health, thus aggravating PD pathology

**Fig. 1.**
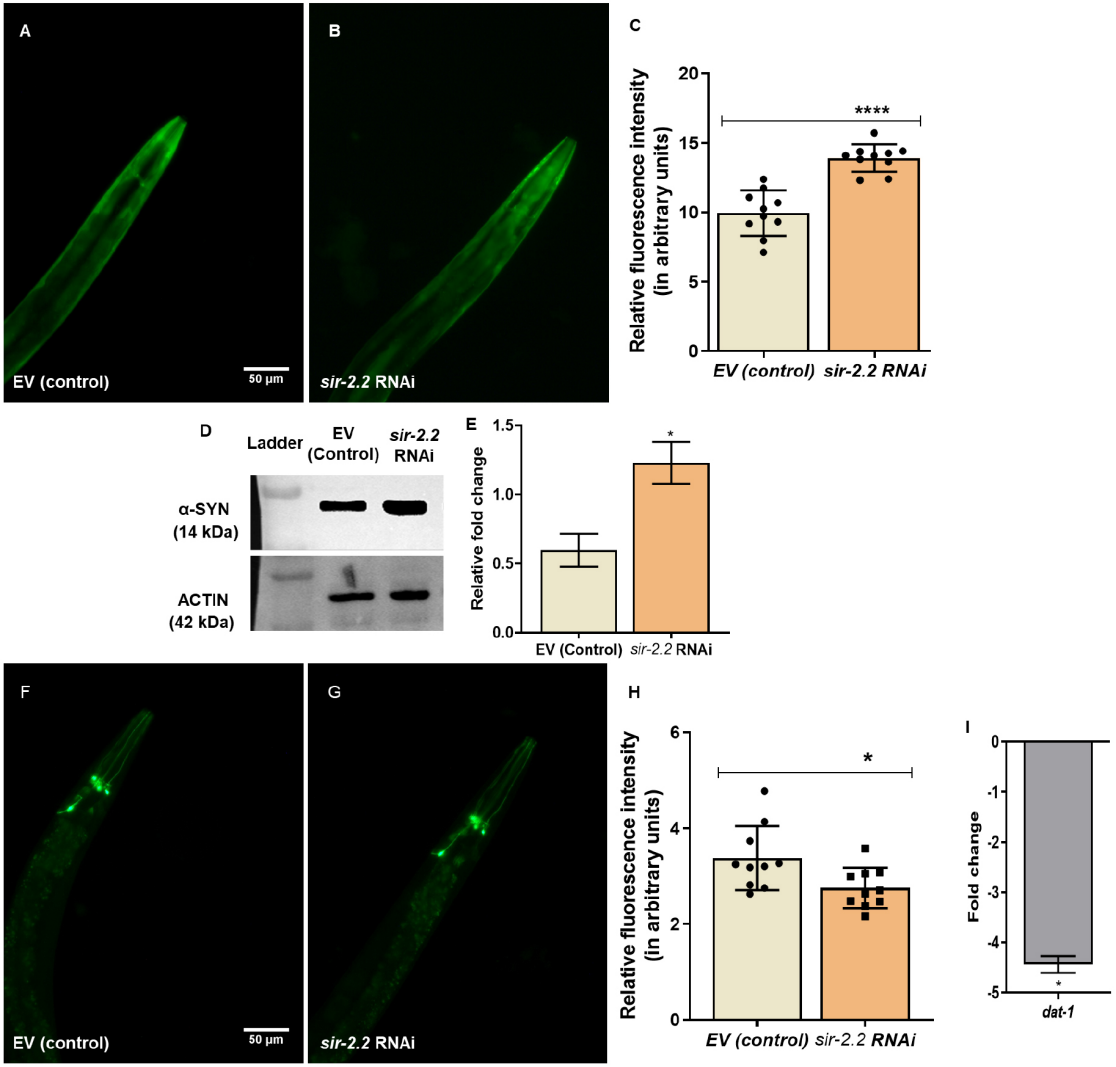
***sir-2.2* knockdown increases alpha-synuclein aggregation and reduces DAT-1 content.** (A,B) Alpha-synuclein aggregation increases after *sir-2.2* gene silencing [by RNA interference (RNAi)] in a model pf Parkinson's disease (PD), the NL5901 strain of *C. elegans*. (A) Transgenic strain NL5901/empty vector (EV) as control group. (B) NL5901/*sir-2.2* gene RNAi. (C) Relative change in fluorescence intensity, suggesting increased alpha-synuclein aggregation after *sir-2.2* gene RNAi. (D) Western blots showing increase in alpha-synuclein protein. (E) Quantification of western blot analysis results. (F,G) DAT-1 expression decreases after *sir-2.2* gene RNAi in BY250 strain. (H) Relative change in fluorescence intensity, suggesting decreased DAT-1 expression after *sir-2.2* gene RNAi. (I) Fold change decrease in mRNA expression levels of *dat-1*, as measured via real-time PCR analysis. Statistical analysis was performed using GraphPad Prism 8 software, employing unpaired two-tailed Student's *t*-test (mean±s.e.m.; *P≤0.05, ****P≤0.0001). Scale bars: 50 µm.

Mitochondrial homeostasis is impaired in neurodegenerative diseases (Jain and Zoncu, 2022). To study mitochondrial homeostasis in neurodegeneration, we employed transgenic strains of *C. elegans* that mimic the PD pathology, such as NL5901 (pkIs2386 [unc-54p::alphasynuclein::YFP+unc-119(+)]). We observed that RNA interference (RNAi)-induced silencing of *sir-2.2* in the NL5901 strain resulted in 1.4-fold increased fluorescence intensity of alpha-synuclein expression (13.91±0.3133) compared to that in the control (9.947±0.5182) (Fig. 1A-C). This result was validated by western blot analysis, which revealed a 2-fold increase in alpha-synuclein protein expression in NL5901 worms with silencing of *sir-2.2* compared to that in controls [empty vector (EV)] (Fig. 1D,E; Fig. S2).

Furthermore, to study dopaminergic neuronal health, we used the BY250 strain (vtIs7 [dat-1p::GFP]). In this strain, fluorescence intensity in *the sir-2.2* KD condition was 2.753±0.1331, compared to 3.375±0.2111 in the control. We observed that DAT-1 expression was decreased 1.3-fold upon *sir-2.2* KD (Fig. 1F-H). The mRNA expression levels of *dat-1* were also reduced 4.4-fold, as shown by real-time PCR analysis (Fig. 1I).

**Fig. 2.**
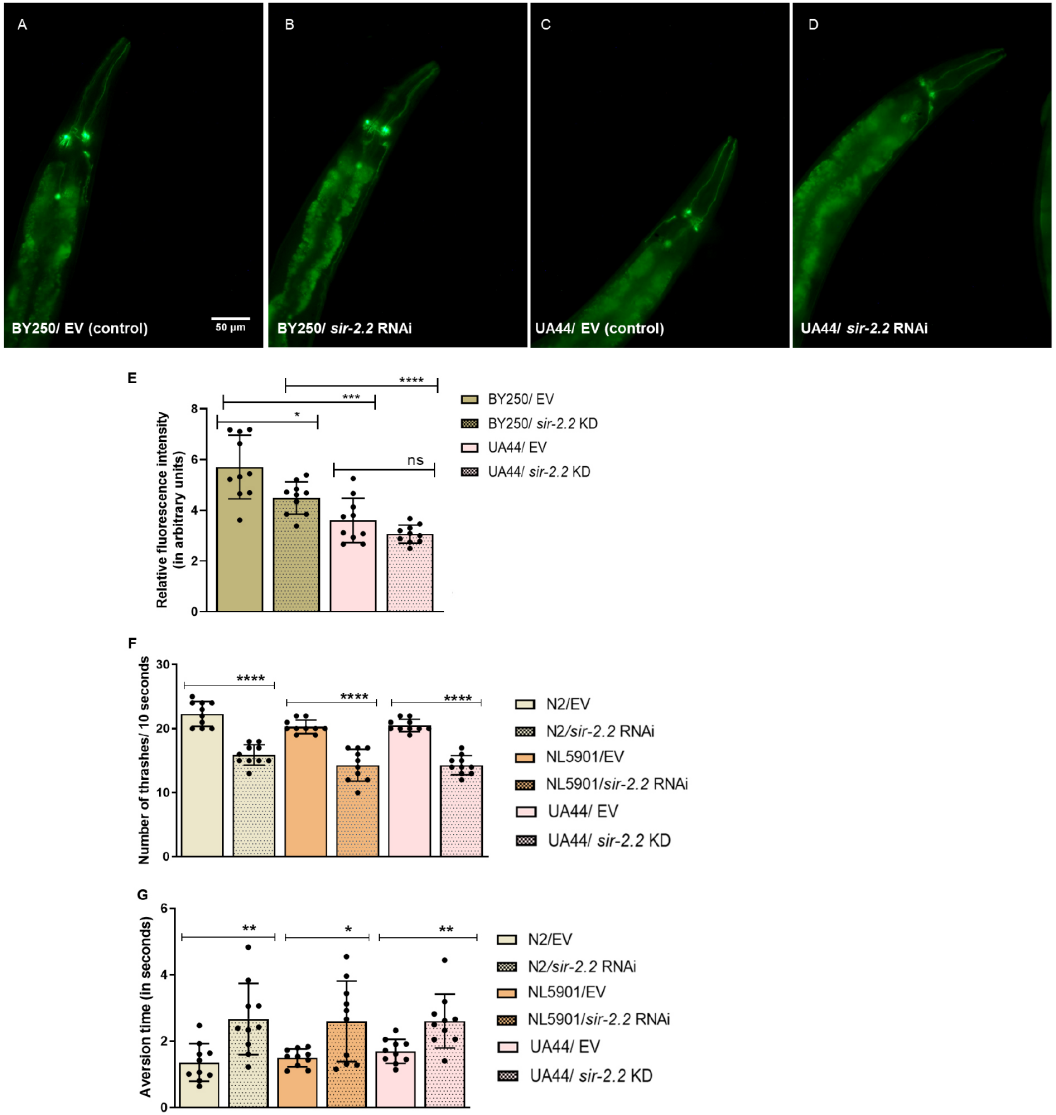
***sir-2.2* knockdown detrimentally affects dopaminergic neuronal health. (**A-D) Degeneration of dopaminergic neurons in the presence of alpha-synuclein, as evident by decreased DAT-1 expression upon *sir-2.2* knockdown in UA44 strain (BY250 as control strain): BY250 strain without gene silencing [empty vector (EV) control; A]; BY250 strain with *sir-2.2* gene silenced (B); UA44 strain without gene silencing (EV control; C); UA44 strain with *sir-2.2* gene silenced (D). (E) Relative change in fluorescence intensity, suggesting decreased DAT-1 expression after *sir-2.2* gene RNAi. (F) Thrashing assay of the number of thrashes per 10 s in worms fed with HT115 bacteria carrying EV as control group, compared to that in worms fed with HT115 bacteria with *sir-2.2* silenced using RNAi, evaluating wild-type N2 strain as well as PD models, NL5901 and UA44 strains. (G) Aversion assay of time taken (in s) to repel from 1-nonanol by worms fed with HT115 bacteria carrying EV as control group, compared to that by worms fed with HT115 bacteria with *sir-2.2* silenced using RNAi, evaluating wild-type N2 strain as well as NL5901 and UA44 strains. Statistical analysis was performed using GraphPad Prism 8 software, employing unpaired two-tailed Student's *t*-test (mean±s.e.m.; ns, non-significant; *P≤0.05, **P≤0.01, ***P≤0.001, ****P≤0.0001). Scale bar: 50 µm.

Next, we assessed the degeneration of dopaminergic neurons in the presence of alpha-synuclein upon *sir-2.2* KD. For this, we employed transgenic strain UA44 (baIn11 [Pdat-1:: α-syn, Pdat-1::GFP]), with the *dat-1* promotor GFP tagged, expressed under the influence of alpha-synuclein protein. We observed a 1.46-fold decrease in dopaminergic neuronal expression at day 7 in UA44 worms upon *sir-2.2* KD compared to that in BY250 worms (Fig. 2A-E). This implied that *sir-2.2* KD decreases dopamine levels.

Dopaminergic neurons play a pivotal role in the movement and odorant responses of *C. elegans* (Pérez-Fernández, Barandela and Jiménez-López, 2021); thus, we found that, upon *sir-2.2* KD, the movement ability of worms was decreased, as the number of thrashes per 10 s was reduced 1.4-fold in N2, 1.4-fold in NL5901 and 1.4-fold in UA44 strains, relative to that in the control group (EV) (Fig. 2F). Also, the aversion response time to 1-nonanol was increased 2-fold in N2, 1.7-fold in NL5901 and 1.5-fold in UA44 strains, relative to that in the control group (EV), suggesting impaired dopaminergic neuronal condition (Fig. 2G).

### *sir-2.2* KD decreases mitochondrial and lipid content

**Fig. 3.**
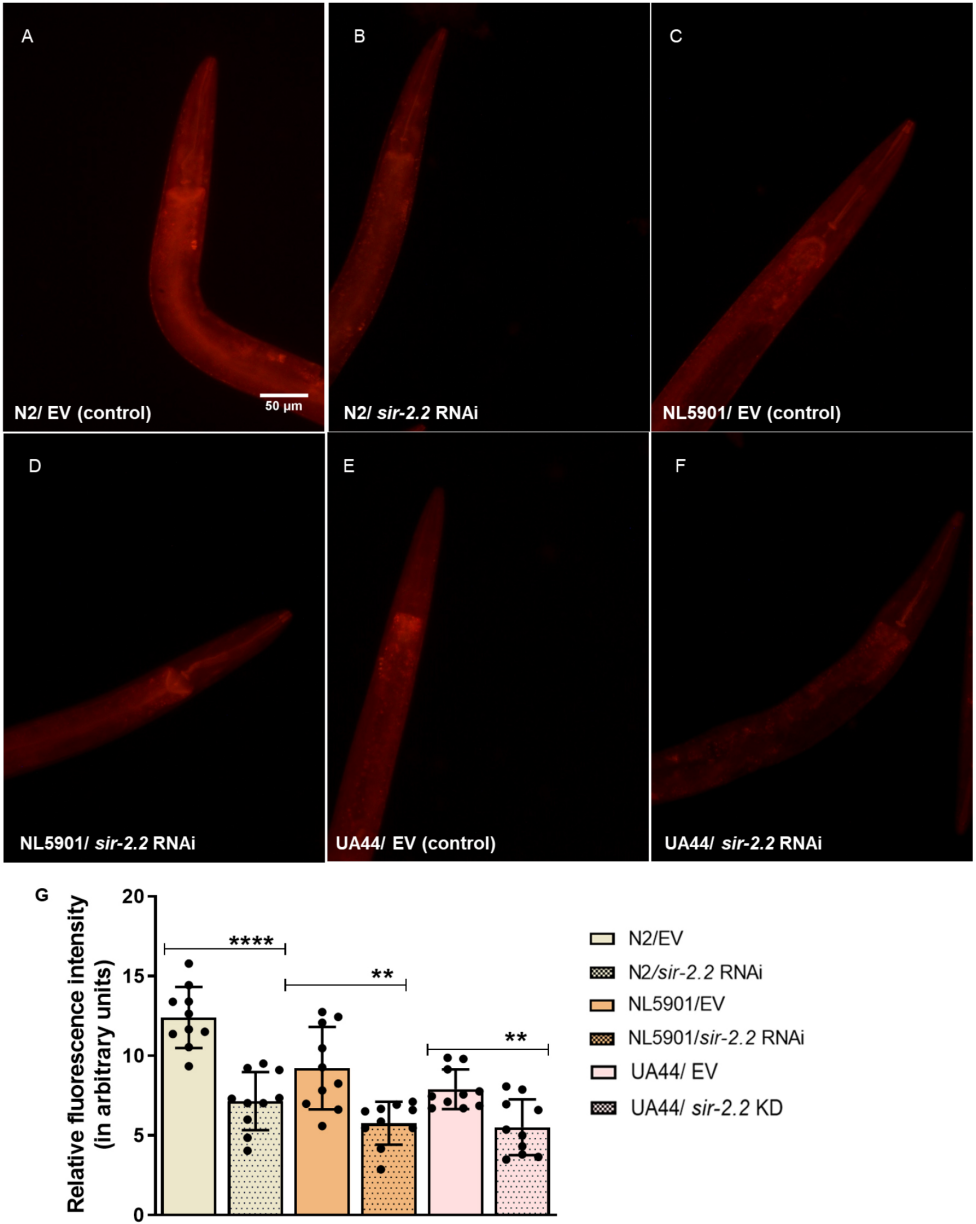
***sir-2.2* knockdown decreases mitochondrial content.** (A-F) Mitochondrial content decreases after *sir-2.2* gene RNAi in wild-type N2 strain (A,B) as well as in PD models, the NL5901 (C,D) and UA44 (E,F) strains of *C. elegans*. (G) Relative change in fluorescence intensity, suggesting decreased mitochondrial content after *sir-2.2* RNAi. Statistical analysis was performed using GraphPad Prism 8 software, employing unpaired two-tailed Student's *t*-test (mean±s.e.m.; **P≤0.01, ****P≤0.0001). Scale bar: 50 µm.

As *sir-2.2* is a mitochondrial protein-coding gene, its KD disrupts mitochondrial biology. Here, we observed that the mitochondrial content was reduced upon *sir-2.2* KD in wild-type N2 (1.7-fold), as well as PD model strains NL5901 (1.6-fold) and UA44 (1.4-fold). This assessment was done using MitoTracker dye, via fluorescence microscopy (Fig. 3A-G).

**Fig. 4.**
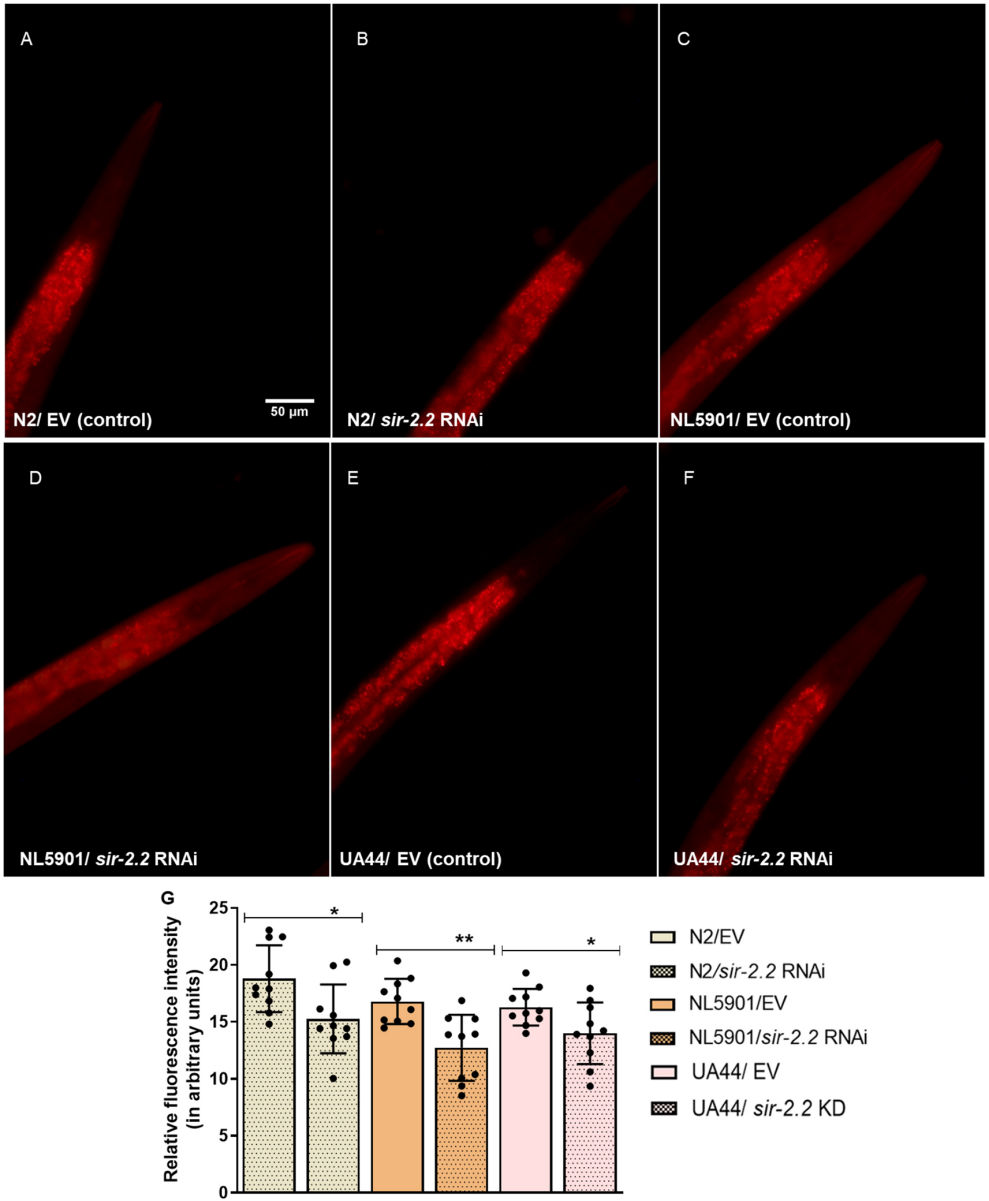
***sir-2.2* knockdown decreases lipid content.** (A-F) Lipid content decreases after *sir-2.2* gene RNAi in wild-type N2 strain (A,B) as well as in PD models, the NL5901 (C,D) and UA44 (E,F) strains of *C. elegans*, via Nile Red dye staining. (G) Relative change in fluorescence intensity, suggesting decreased lipid content after *sir-2.2* RNAi. Statistical analysis was performed using GraphPad Prism 5 software, employing unpaired two-tailed Student's *t*-test (mean±s.e.m.; *P≤0.05, **P≤0.01). Scale bar: 50 µm.

Besides reducing mitochondrial content, *sir-2.2* KD reduced the lipid content of worms, as measured using Nile Red dye via fluorescence microscopy in wild-type N2 (1.2-fold), as well as PD model strains NL5901 (1.3-fold) and UA44 (1.3-fold) (Fig. 4A-G).

### *sir-2.2* KD disrupts mitochondrial homeostasis by increasing reactive oxygen species (ROS) production and reducing the expression of mitochondrial biology markers

**Fig. 5.**
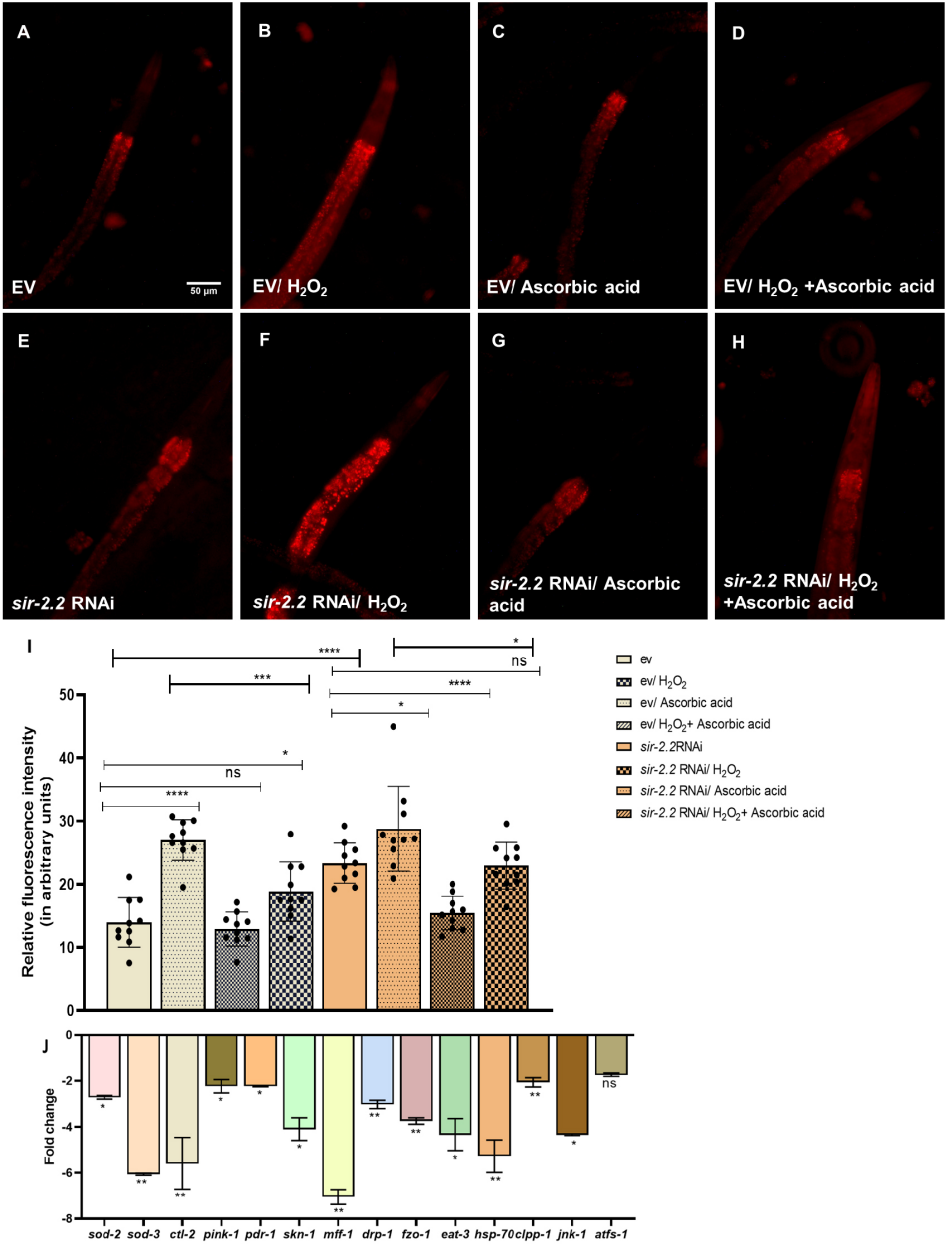
***sir-2.2* knockdown disrupts mitochondrial homeostasis.** (A-H) Reactive oxygen species (ROS) estimation using dihydroethidium (DHE) dye (red). (I) Quantification of ROS levels. Basal as well as induced ROS increase upon *sir-2.2* gene knockdown, in wild-type N2 strain of *C. elegans*. (J) Quantitative PCR analysis of mRNA expression levels of mitochondrial biology markers of antioxidant activity (*sod-2*, *sod-3*, *ctl-2*), stress response (*skn-1*), mitophagy (*pink-1*, *pdr-1*), mitochondrial fission (*mff-1*, *drp-1*), mitochondrial fusion (*fzo-1*, *eat-3*) and mitochondrial unfolded protein response (*hsp-70*, *clpp-1*, *jnk-1*, *atfs-1*). Statistical analysis was performed using GraphPad Prism 8 software, employing unpaired two-tailed Student's *t*-test (mean±s.e.m.; ns, non-significant; *P≤0.05, **P≤0.01,***P≤0.001, ****P≤0.0001). Scale bar: 50 µm.

*sir-2.2* KD increased the basal ROS, by 1.6-fold, and the induced ROS using H_2_O_2_, by 1.06-fold, which was rescued to a 1.25-fold increase upon administration of antioxidant ascorbic acid (30 mM), as estimated using dihydroethidium (DHE) dye in the wild-type N2 strain (Fig. 5A-I).

Because mitochondrial autophagy, also known as mitophagy, and unfolded protein response (UPR) are important aspects of governing the proper clearance of unwanted proteins, they are also important for maintaining homeostasis (Li et al., 2022; Suárez-Rivero et al., 2022). Thus, we studied the mRNA expression levels of genes associated with different aspects of mitochondrial homeostasis and energy metabolism, such as markers of antioxidant activity (*sod-2*, *sod-3*, *ctl-2*), stress response (*skn-1*), mitophagy (*pink-1*, *pdr-1*), mitochondrial fission (*mff-1*, *drp-1*), mitochondrial fusion (*fzo-1*, *eat-3*) and mitochondrial UPR (*hsp-70*, *clpp-1*, *jnk-1*, *atfs-1*), upon *sir-2.2* KD and found that these levels were reduced by 2.7-, 6-, 5.6-, 4.1-, 2.2-, 2.2-, 7.1-, 3-, 3.7-, 4.3-, 5.2-, 2-, 4.3- 1.7-fold, respectively, upon *sir-2.2* KD (Fig. 5J).

### *sir-2.2* KD impairs energy metabolism by decreasing ATP levels and mitochondrial membrane potential, suggesting induction of mitochondrial membrane depolarization

**Fig. 6.**
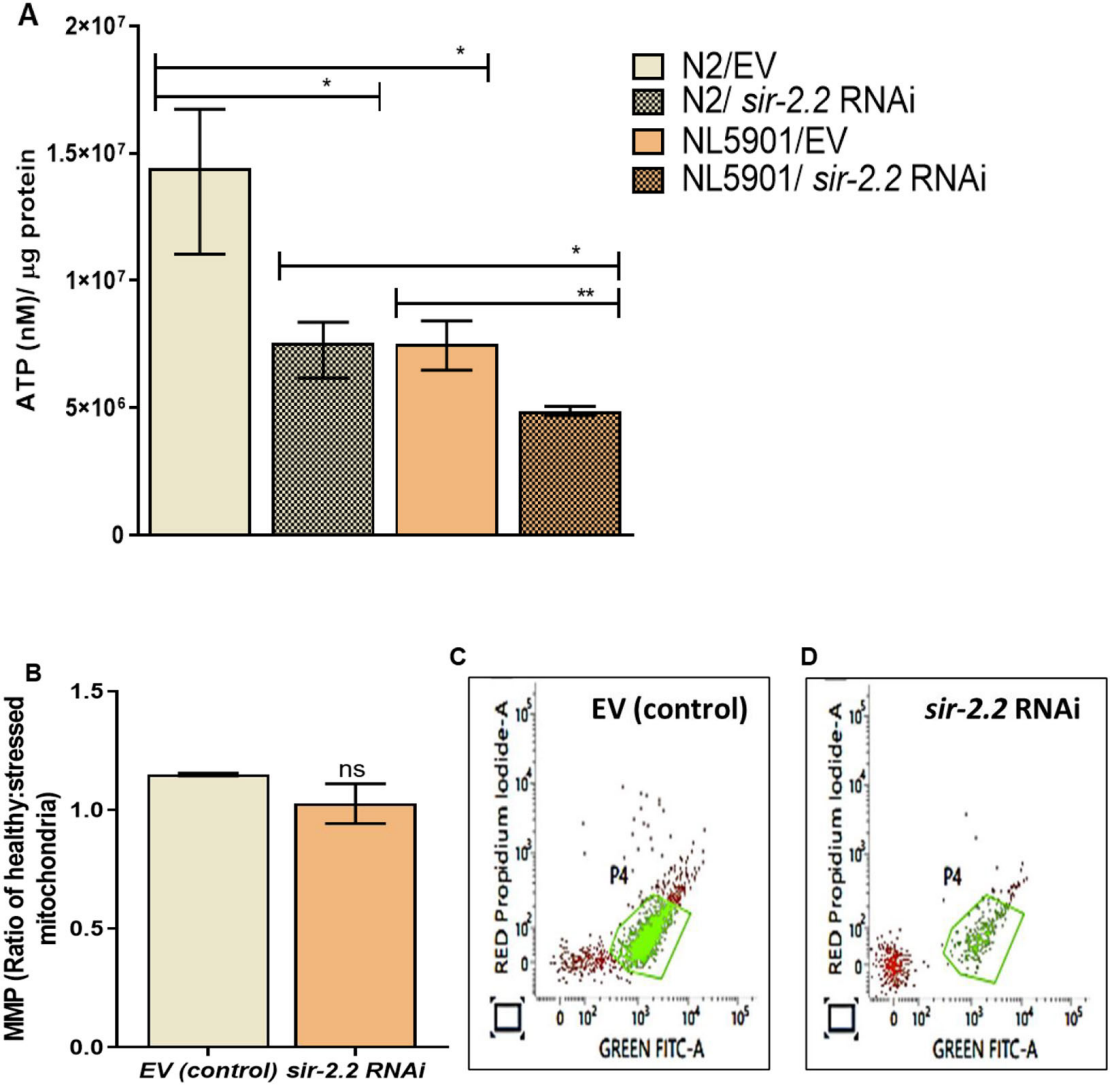
***sir-2.2* knockdown impairs energy metabolism.** (A) ATP levels in N2 and NL5901 strain upon *sir-2.2* knockdown. (B-D) Mitochondrial membrane potential (MMP) analysis showing reduced MMP levels upon *sir-2.2* knockdown in N2 strain. (C) Graph depicting cells with red versus green fluorescence in control group (EV). (D) Graph depicting cells with red versus green fluorescence in *sir-2.2* RNAi group. In C and D, propidium iodide was used as a marker of the integrity of the cell membrane. Statistical analysis was performed using GraphPad Prism 8 software, employing unpaired two-tailed Student's *t*-test (mean±s.e.m.; ns, non-significant; *P≤0.05, **P≤0.01).

To understand mitochondrial energy metabolism in detail, we endeavoured to study the effect of *sir-2.2* KD on energy homeostasis. Thus, we estimated the levels of ATP production using an ATP bioluminescent assay kit. We found that the levels of ATP were significantly reduced 1.9-fold in wild-type N2 and 1.5-fold in transgenic NL5901 worms compared to those in their respective controls (Fig. 6A).

We further assessed the role of *sir-2.2* in regulating mitochondrial membrane potential (MMP) using JC-1 dye. We observed that there was a non-significant reduction (0.89-fold) in the ratio of healthy: stressed mitochondria, suggesting induction of mitochondrial membrane depolarization (Fig. 6B-D).

### *sir-2.2* KD disrupts autophagy and innate immunity

**Fig. 7.**
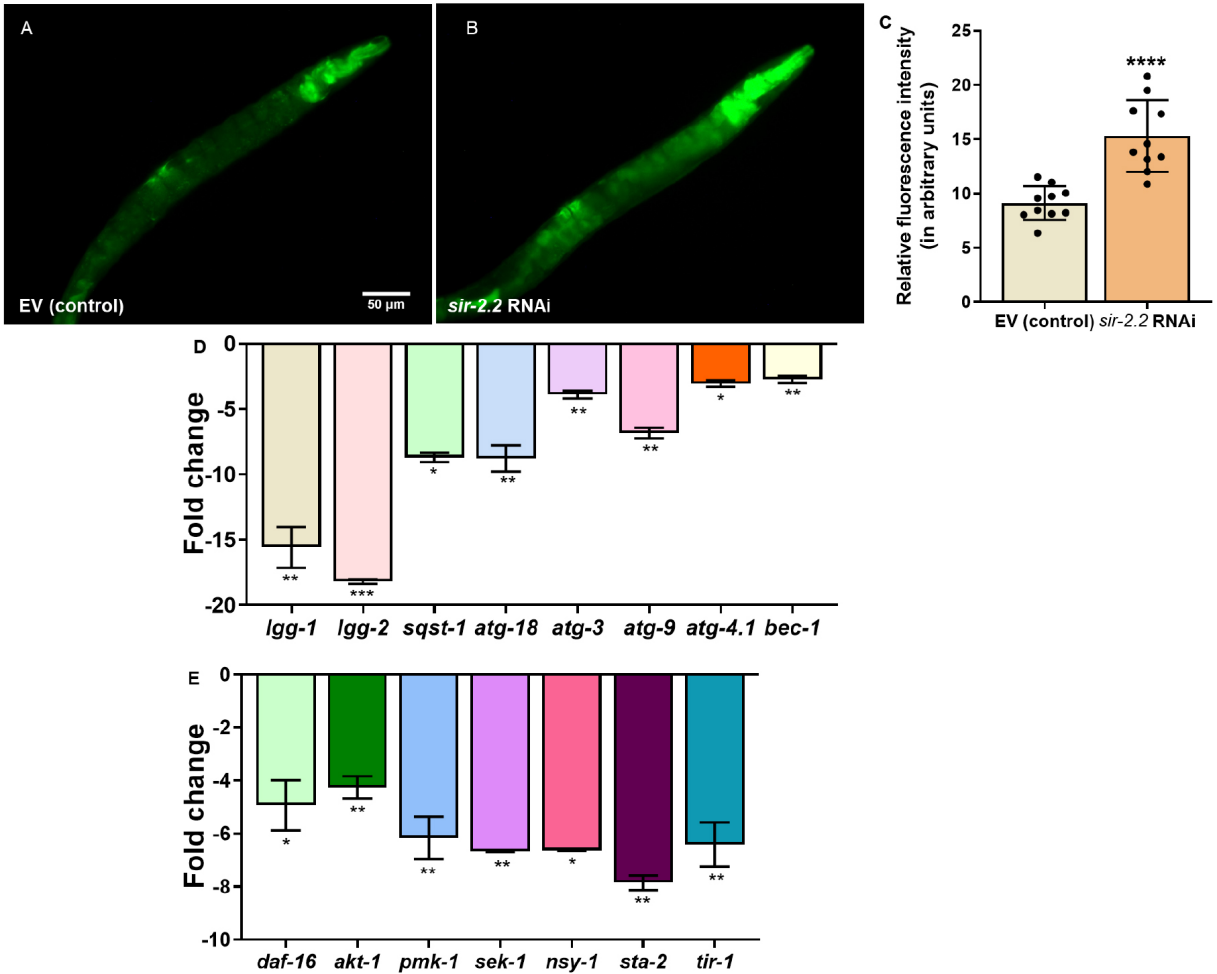
***sir-2.2* knockdown disrupts autophagy and innate immunity.** (A,B) Accumulation of autophagy marker SQST-1 upon *sir-2.2* gene knockdown in transgenic strain BC12921 (B; compared to EV control in A), suggesting impaired clearance during autophagy. (C) Relative change in fluorescence intensity after *sir-2.2* gene silencing. (D,E) Quantitative PCR analysis of mRNA expression levels of autophagy markers such as *lgg-1*, *lgg-2*, *sqst-1*, *atg-18*, *atg-3*, *atg-9*, *atg-4.1* and *bec-1* (D), and innate immune response markers such as *daf-16*, *akt-1*, *pmk-1*, *sek-1*, *nsy-1*, *sta-2* and *tir-1* (E), in wild-type N2 strain of *C. elegans.* Here, the fold change values depict the normalized values (normalized to control). Statistical analysis was performed using GraphPad Prism 5 software, employing unpaired two-tailed Student's *t*-test (mean±s.e.m.; *P≤0.05, **P≤0.01, ***P≤0.001, ****P≤0.0001). Scale bar: 50 µm.

Protein build-up is one of the pathologies of neurodegeneration; thus, impaired autophagy machinery is a cause of disease progression. Here, we assessed the role of *sir-2.2* in regulating autophagy. First, we employed a well-studied marker strain for autophagy, BC12921, which has *sqst-1*, an orthologue of the gene encoding the human autophagy receptor p62 (also known as SQSTM1), tagged with GFP. We found accumulation of SQST-1 protein under the *sir-2.2* KD condition, with worms showing a 1.68-fold increase in fluorescence intensity relative to that in controls, suggesting disturbed protein clearance (Fig. 7A-C). Next, we examined the mRNA expression levels of autophagy markers *lgg-1*, *lgg-2*, *sqst-1*, *atg-18*, *atg-3*, *atg-9*, *atg-4.1* and *bec-1*, which were found to be reduced by 15.5-, 18.1-, 8.7-, 8.7-, 3.8-, 6.8-, 3- and 2.7-fold, respectively (Fig. 7D).

Because silencing of *sir-2.2* resulted in impeded autophagic response, we investigated the role of *sir-2.2* in the regulation of innate immunity. We evaluated the mRNA expression levels of innate immune response marker genes such as *daf-16*, *akt-1*, *pmk-1*, *sek-1*, *nsy-1*, *sta-2* and *tir-1*, observing that *sir-2.2* KD decreased *daf-16*, *akt-1*, *pmk-1*, *sek-1*, *nsy-1*, *sta-2* and *tir-1* expression by 4.9-, 4.3-, 6.2-, 6.7-, 6.6-, 7.9- and 6.4-fold, respectively (Fig. 7E). These results suggest that *sir-2.2* plays an important role in modulating PD pathology via autophagy and innate immunity.

## DISCUSSION

In this study, we investigated the role of mitochondrial sirtuins in regulating age-related neurodegenerative disorders, particularly PD. Given the central role of mitochondria as the primary energy-producing organelle within a cell, we focused on how mitochondrial sirtuins influence neurodegeneration and used an alpha-synuclein-expressing *C. elegans* model for the investigations. Over the past decade, mitochondria have garnered significant attention for their pivotal function in cellular metabolism and their emerging potential as therapeutic targets for neurodegenerative diseases (Petkovic et al., 2021; Vrijsen et al., 2022; Collier et al., 2023). Mitochondria are known to play a significant role in regulating an organism's homeostasis (Yu and Pekkurnaz, 2018; Casanova et al., 2023) and, thus, are important for keeping many age-associated diseases at bay (Trigo et al., 2022; Li et al., 2024). Ageing and neurodegeneration go hand in hand, and numerous studies have suggested that ageing is one of the major reasons for the development of neurological disorders (Hou et al., 2019; Gonzales et al., 2022). Thus, we explored the role of sirtuins, the most widely studied class of longevity proteins, in neurodegeneration. Sirtuins, predominantly the mitochondrial sirtuins, are involved in regulating mitochondrial homeostasis, cellular stress response and redox balance; thus, they play a critical role in governing the occurrence and progression of various diseases, including neurodegenerative disease such as PD, in which alpha-synuclein aggregation leads to mitochondrial dysfunction. Human mitochondrial sirtuins such as SIRT3 are involved in mitochondrial metabolism and insulin sensitivity (Ji et al, 2022); SIRT3, along with SIRT5, plays a role in regulating fasting-induced fatty acid oxidation by modulating AMPK activity. Studies have suggested a therapeutic role for mitochondrial sirtuins in regulating metabolic disorders (Wang and Wei, 2020). In *C. elegans*, mitochondrial sirtuins are encoded by *sir-2.2* and *sir-2.3* (Viswanathan and Tissenbaum, 2013). *sir-2.2* is an orthologue of human *SIRT4*. Its encoded protein exerts NAD^+^-dependent histone deacetylase activity and is involved in regulating energy homeostasis, cellular survival and innate immune functions; however, its role in neuroprotection has not been studied extensively (Wirth et al., 2013). Although *sir-2.1* has been widely studied for its role in regulating neuroprotection (Donmez, 2012; Chandramowlishwaran et al., 2020; Yeong et al., 2020; Naseer et al., 2021), the involvement of *sir-2.2* is not established. As *sir-2.2* is expressed in the nervous system, hypodermis, excretory system, reproductive system and non-striated muscles, we hypothesized that it might have a prominent role in regulating neurodegeneration. Thus, we employed the RNAi technique via feeding for silencing *sir-2.2* to evaluate its effects in modulating neurodegeneration.

Sirtuins possess strong deacetylation activity, whereby they remove the acetyl group of alpha-synuclein at lysine 6 and lysine 10 positions, thereby modulating protein aggregation and clearance (de Oliveira et al., 2017). Hence, we first validated the downregulation of *sir-2.2* using real-time PCR (Fig. S3). Next, we assessed the role of *sir-2.2* in PD pathology by employing transgenic strains NL5901, UA44 and BY250. We observed that, upon *sir-2.2* KD, the level of alpha-synuclein aggregation was increased, as evidenced by imaging and western blot analysis.

We also found that *sir-2.2* KD results in disruption of dopaminergic neurons, as measured via *dat-1* gene downregulation through real-time PCR and DAT-1 expression via fluorescence imaging. Additionally, upon *sir-2.2* KD, different behavioural parameters that were well associated with dopaminergic neuronal health, such as motility and sense of smell toward odorants such as 1-nonanol, were affected in wild-type as well as PD condition.

Subsequently, our research showed that KD of *sir-2.2* markedly affects the mitochondrial biology, showing its critical role in energy metabolism. This was evident by a decrease in the mitochondrial, as well as lipid, content when measured by MitoTracker and Nile Red dyes, respectively, in wild-type N2 as well as PD models NL5901 and UA44. We found that *sir-2.2* KD results in the disruption of oxidative stress response (Wirth et al., 2013; Dilberger et al., 2019) by decreasing the levels of superoxide dismutase (*sod-2*, *sod-3*) and catalase (*ctl-2*) genes and also by increasing ROS production. Mitochondria are exclusively involved in the regulation of stress response pathways, such as heat (Labbadia et al., 2017; Adriaenssens et al., 2023) and mitochondrial unfolded protein response (UPR^mt^) (Muñoz-Carvajal and Sanhueza, 2020; Suárez-Rivero et al., 2022; Wodrich et al., 2022). NAD^+^ being a co-factor in SIR-2.2 activity suggests its potential to sense energy fluctuations, which further activates UPR^mt^ machinery to combat stressful conditions (Liu et al., 2024; Morrow et al., 2024). The UPR^mt^ machinery is crucial for normal functioning of the body, and, in several diseases such as PD, this machinery gets disturbed and results in the manifestation of disease pathology (Zhu et al., 2021; Chen et al., 2023). We investigated the effect of *sir-2.2* KD on the expression of well-established UPR^mt^ markers, such as *hsp-70*, *clpp-1*, *jnk-1* and *atfs-1* (Bennett et al., 2014), observing a significant decrease in the expression of these UPR^mt^ markers, suggesting disturbance in the protein-folding machinery upon *sir-2.2* KD.

Mitochondrial health is also controlled by a crosstalk between mitochondria and autophagy machinery (Rambold and Lippincott-Schwartz, 2011; Li et al., 2022). Mitochondrial autophagy, also known as mitophagy, is the process by which a cell clears itself of damaged mitochondria (Youle et al., 2014). It forms an active part of the protein clearance machinery, thus maintaining homeostasis. Thus, we investigated the effect of *sir-2.2* KD on mitophagy machinery and found that mitophagy is highly disrupted upon *sir-2.2* KD, as evidenced by a decrease in mitophagy markers *pink-2* and *pdr-1.*

Mitochondria are an integral part of energy homeostasis, and our observations of mitochondrial *sir-2.2* KD resulting in reduced ATP levels in N2 as well as in NL5901 suggest compromised energy utilization in PD pathology. ATP production relies on the polarity of the MMP generated during oxidative phosphorylation via electron transport in the electron transport system (Sarkar et al., 2022). Because mitochondrial sirtuins do not directly impair glycolysis, literature suggests that their KD can lead to increased sensitivity to oxidative stress, affecting oxidative phosphorylation (Wirth et al., 2013). This further leads to a buildup of ROS, hampering cellular metabolism, as evidenced by our results.

As mitochondrial *sir-2.2* is known to enable NAD^+^ binding activity together with NAD-dependent histone deacetylase activity and is known to be directly participating in proton pumping across the mitochondrial inner membrane to establish a proton gradient during electron transport, we investigated MMP in *sir-2.2* KD condition. However, our results indicate that RNAi of *sir-2.2* does not significantly alter the MMP. Autophagy is also an important mechanism in modulating immunity, as evidenced by various studies (Levine et al., 2011; Germic et al., 2019; Jiang et al., 2019; Pradel et al., 2020) and *sir-2.2* being an important regulator of the innate immune response; we observed that *sir-2.2* KD impairs the autophagy machinery and decreases the mRNA expression levels of innate immune response marker genes such as *daf-16*, *akt-1*, *pmk-1*, *sek-1*, *nsy-1*, *sta-2* and *tir-1*.

**Fig. 8.**
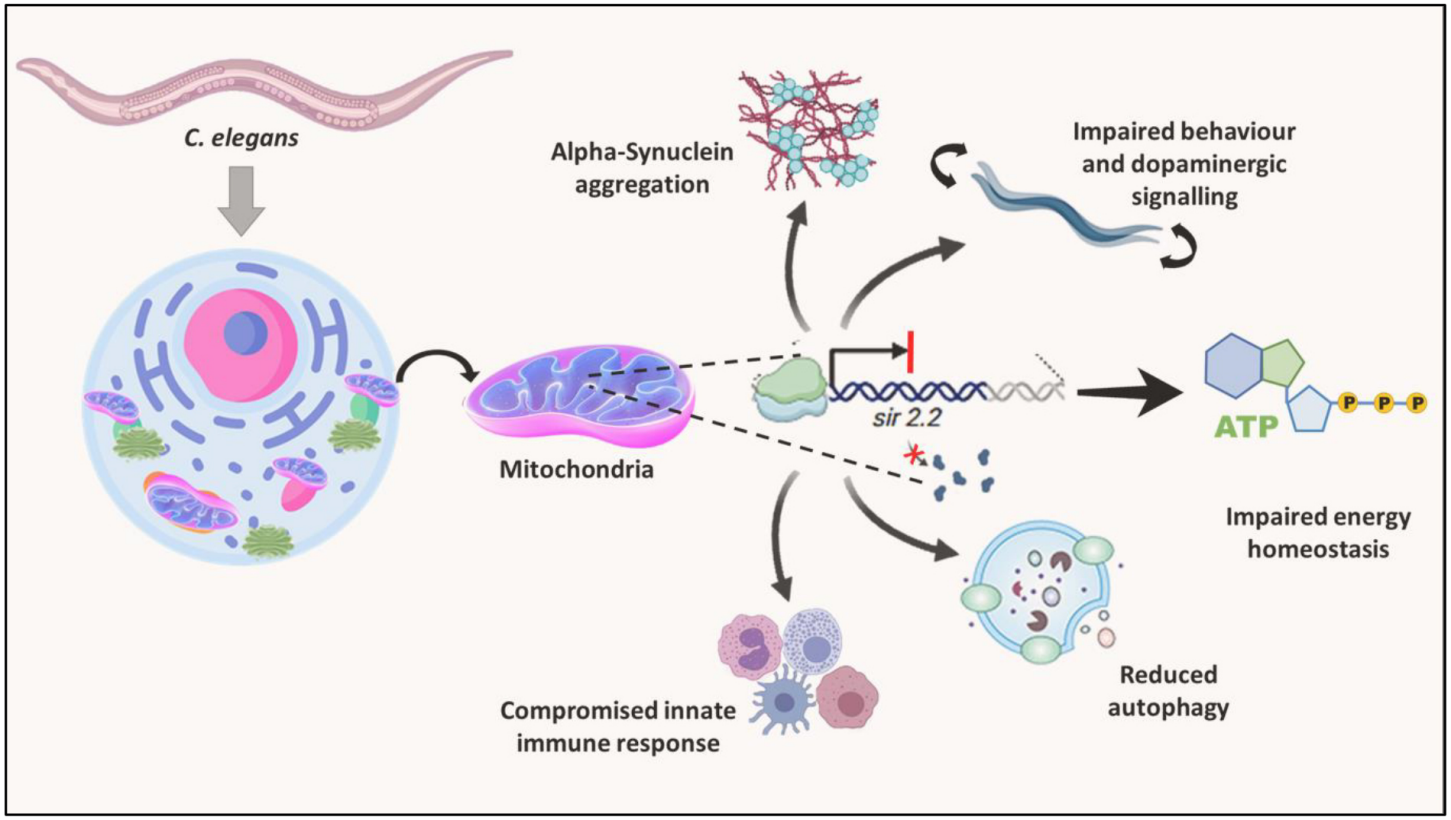
Graphical summary.

These findings highlight the critical role of the mitochondrial sirtuin SIR-2.2 in neuroprotection and mitochondrial homeostasis (Fig. 8), advancing our understanding of the mechanistic aspects of age-associated neurodegenerative diseases. As sirtuins are pathophysiologically and physiologically active, they have potential as therapeutic targets; for example, sirtuin activators such as resveratrol are known for lifespan extension effects (Bhullar and Hubbard, 2015; Song et al., 2021) and sirtuin inhibitors such as sirtinol have been well studied for their anti-tumorigenic activity (Hu et al., 2014). Similarly, mitochondrial sirtuins, especially SIRT3, interact with mTOR regulators and inhibit tumour progression, also sensing the nutrient deficiency via NAD^+^ and activating the AMPK pathway to restore balance (Zhang et al., 2020). Thus, these proteins are promising molecules for designing therapeutics. Further in-depth studies to elucidate the detailed mechanisms underlying mitochondrial quality control and efficient protein clearance in neurodegenerative disorders will bring us closer to leveraging these pathways for successful translational applications.

## MATERIALS AND METHODS

### *C. elegans* strains and worm culture

*C. elegans* strains used in this study include wild-type N2 (Bristol) and transgenic strains such as NL5901 (pkIs2386 [unc-54p::alphasynuclein::YFP+unc-119(+)]), BY250 (vtIs7 [dat-1p::GFP]), UA44 (baIn11 [Pdat-1:: α-syn, Pdat-1::GFP]) and BC12921 (sIs10729 [rCes T12G3.1::GFP+pCeh361]). These strains were cultured using standard protocols. Worms were fed *Escherichia coli* strain OP50 and grown on nematode growth medium (NGM), prepared by adding 50 mM sodium chloride (HIMEDIA; MB023), 2.5 g peptone (HIMEDIA; RM001) and 17 g agar (HIMEDIA; GRM026P) in 975 ml double-distilled water and autoclaved at 121°C for 45 min at 15 psi. Then, the medium was cooled to 50-60°C, and 5 mg ml^−1^ cholesterol (Sigma-Aldrich; C75209) solution (dissolved in ethanol), 1 M calcium chloride (HIMEDIA; MB034), 1 M magnesium sulphate (MP Biomedicals; 194699) and 25 l potassium dihydrogen phosphate (Sigma-Aldrich; P5655) were added. To prepare isopropyl β-d-1-thiogalactopyranoside (IPTG)-NGM plates for RNAi experiments, 23.83 mg ml^−1^ IPTG (Sigma-Aldrich; I6758) in milli-Q water (MQ) and 2.5 mg ml^−1^ carbenicillin (Sigma-Aldrich; C1389) in MQ were added to the NGM reagents, mixed and poured into Petri dishes. The contents of the Petri dishes were then allowed to solidify and were stored at 4°C.

Standard conditions were followed for *C. elegans* propagation. All experiments were carried out at 22°C in a Thermo Fisher Scientific Lab-Tech B.O.D. Incubator (Brenner, 1974; Tissenbaum, 2015). Synchronized worms were obtained by embryo isolation protocol. Briefly, worms were washed with M9 buffer and axenized using 2 ml sodium hypochlorite (Sigma-Aldrich; 239305) and 5 ml of 1 M sodium hydroxide (Qualigens; Q27815) solution, and the released eggs were seeded onto the Petri dishes.

#### *E. coli* strain OP50 culture

For preparation of 100 ml *E. coli* OP50 (uracil auxotroph) culture, 300 mg KH_2_PO_4_ (Sigma-Aldrich; P5655), 600 mg Na_2_HPO_4_ (Sigma-Aldrich; S9763) and 500 mg NaCl (HIMEDIA; MB023) were added to distilled water and autoclaved at 121°C for 45 min at 15 psi. Then, 200 µl uracil (MP Biomedicals; 194761) (2 mg ml^−1^ in MQ), 1 ml dextrose (SRL; 0449130) (200 mg ml^−1^ in MQ), 100 µl MgSO_4_ (MP Biomedicals; 194699), 1 ml NH_4_Cl (Sigma-Aldrich; A9434) and 500 µl *E. coli* OP50 inoculum were added, before incubation at 37°C overnight on an incubator-shaker (New Brunswick Scientific Innova42, Incubator shaker series) at 180 rpm. Approximately 500 µl *E. coli* OP50 culture (inoculum) was seeded on previously prepared NGM plates in a Bio-Safety Cabinet (Thermo Fisher Scientific; 1300 series A2) to create a uniform lawn, before incubation overnight at 22°C (Corsi et al., 2015).

#### Measurement of lipid content using Nile Red dye

To investigate the effect of *sir-2.2* gene KD on lipid content, we employed a Nile Red staining protocol (Jadiya et al., 2016). Briefly, the stage-synchronous worms were pre-stained with Nile Red dye (MP Biomedicals; 151744) starting at embryo stage. After 48 h, the young-adult worms were washed with M9 buffer three times to remove excess adherent bacteria and were immobilized using 100 mM sodium azide (Sigma-Aldrich; 71289) solution for 5 min. Approximately 20 µl of the sample was placed onto glass slides, and images were taken using a Rhodamine filter on a Carl Zeiss Axio Imager.M2 microscope. Fluorescence intensity was quantified using ImageJ software (National Institutes of Health, Bethesda, MD, USA) (*n*=10; three replicates).

#### Measurement of mitochondrial content using MitoTracker dye

To investigate the effect of *sir-2.2* gene KD on mitochondrial content, we employed a MitoTracker dye staining protocol (Jadiya et al., 2016). Briefly, the stage-synchronous worms were pre-stained with MitoTracker dye (Invitrogen; MitoTracker TM Red CMX Ros, M7512) starting at embryo stage. After 48 h, the young-adult worms were washed with M9 buffer three times to remove excess adherent bacteria and were immobilized using 100 mM sodium azide (Sigma-Aldrich; 71289) solution for 5 min. Approximately 20 µl of the sample was placed onto glass slides, and images were taken using a Rhodamine filter on a Carl Zeiss Axio Imager.M2 microscope. Fluorescence intensity was quantified using ImageJ software (*n*=10; three replicates).

#### Fluorescence imaging

Briefly, the stage-synchronised young-adult worms were washed three times with M9 buffer to remove excess adherent bacteria and were immobilized using 100 mM sodium azide (Sigma-Aldrich; 71289) solution for 5 min. Approximately 20 µl of the sample was placed onto glass slides, and images were taken using a specific filter on a Carl Zeiss Axio Imager.M2 microscope. The fluorescence intensity of alpha-synuclein and DAT-1 expression was quantified using ImageJ software (*n*=10; three replicates).

#### ATP assay

ATP levels under the *sir-2.2* KD condition were determined using an ATP Bioluminescent Assay Kit (Sigma-Aldrich; FLAA) according to a well-established protocol (Sarkar et al., 2022). Briefly, ∼300 worms were pelleted down from control and *sir-2.2* KD groups of N2 wild-type strain, and protein isolation was performed as described in the ‘Western blotting’ section. Protein concentration was measured using a Pierce™ BCA Protein Assay Kit (Thermo Fisher Scientific; 23225). ATP was measured by adding ATP assay solution to the protein sample and ATP dilution buffer mixture and measuring the ATP levels using the light emitted in a luminometer (Promega GloMax^®^). Statistical analysis was performed using GraphPad Prism 8 software, employing unpaired two-tailed Student's *t*-test.

#### 1-nonanol assay

In *C. elegans*, the 1-nonanol assay provides information regarding dopaminergic neuronal health. 1-nonanol is an odour repellent, and the time taken for repulsion after coming into its vicinity determines the dopaminergic neuronal health of the worms (Jadiya et al., 2016). Briefly, control and *sir-2.2* gene-silenced worms were grown on IPTG-NGM plates and were synchronized using embryo isolation. After 48 h, the worms were washed three times with M9 buffer to remove excess adherent bacteria, and one worm at a time was placed on a glass slide for analysis using a Leica S8AP0 stereo-zoom microscope at 1× magnification. Then, a rod dipped in 1-nonanol (MP Biomedicals; 216403) was brought near the snout of the worm, and repulsion time was noted. The level of significance was calculated for ten worms per group using GraphPad Prism 8 software, employing unpaired two-tailed Student's *t*-test.

#### Thrashing assay

The thrashing assay involves the measurement of the motility rate of worms. In *C. elegans*, this assay provides information regarding the level of dopamine content, which controls worms' motility (Jadiya et al., 2016). Briefly, control and *sir-2.2* gene-silenced worms were grown on IPTG-NGM plates and synchronized using embryo isolation. After 48 h, the worms were washed three times with M9 buffer to remove excess adherent bacteria, Then, a drop of M9 buffer was placed on a glass slide, and the number of thrashes (movement of body from one side of the body to the opposite side and back to original side) after every 10 s for each worm, one worm at a time, was counted using a Leica S8AP0 stereo-zoom microscope at 1× magnification. The mean number of thrashes was calculated, and the level of significance was determined for ten worms per group, using GraphPad Prism 8 software, employing unpaired two-tailed Student's *t*-test.

#### RNAi

Gene silencing was carried out employing the feeding protocol described previously (Jadiya et al., 2016). HT115 bacterial clone-producing dsRNA against *sir-2.2* was obtained from the Ahringer RNAi library (purchased from SA Biosciences, Cambridge, UK) and was cultured for 6-8 h in Luria-Bertani (LB) broth containing ampicillin (50 µg ml^−1^). This LB-bacterial culture was seeded onto IPTG-NGM plates and incubated overnight at 22°C. For experiments, embryos were transferred onto these plates to obtain age-synchronous worms.

#### Quantitative real-time PCR (qPCR) analysis for mRNA expression of genes

For this, we carried out RNA isolation of the control and *sir-2.2* gene-silenced worms followed by cDNA preparation and mRNA quantification using qPCR. Briefly, young-adult worms were harvested in M9 buffer and then washed three times with 0.2% DEPC-treated water (Sigma-Aldrich; D5758). RNAzol (Molecular Research Center; RN190) was used to isolate RNA, following a standard protocol (Shukla et al., 2021 preprint). The total RNA isolated was then subjected to cDNA synthesis using a verso cDNA synthesis kit (Thermo Fisher Scientific; AB-1453/B) and PCR (Agilent Technologies; Sure Cycler 8800). qPCR analysis (Agilent Technologies; Stratagene Mx3005P) was done using 100 ngµl^−1^ cDNA [measured using a spectrophotometer (Thermo Fisher Scientific; NANO DROP ONE^C^)] employing Takara Master Mix (TB Green^®^ Primer Ex Taq^TM^, RR420A), and quantification was done by normalizing to actin as the reference gene. The PCR cycle consisted of (1) one cycle of pre-incubation: 95°C for 30 s; and (2) 40 cycles of amplification: 95°C for 5 s, 55°C for 30 s, 60°C for 35 s. The obtained Ct values were processed further and normalized with the control values, and relative fold change in gene expression was quantified using the 2^–ΔΔCt^ method (Table S1).

#### Western blotting

To validate that *sir-2.2* KD increases the expression of alpha-synuclein protein, we first prepared the protein sample and then performed SDS-PAGE followed by western blotting. Briefly, young-adult worms of the control and *sir-2.2* gene-silenced condition were washed three times with M9 buffer to remove excess adherent bacteria and were re-suspended in (1×) PBS. The worms were sonicated in (1×) PBS using a sonicator (Haatman; JY92-IIN) at 25% amplitude for 3 min (pulse time ON/OFF, 30 s). The lysate was then centrifuged at 13,000 ***g*** for 20 min at 4°C. The supernatant was transferred to a fresh micro-centrifuge tube containing the total protein, while debris was discarded. Protein concentration was calculated using a Pierce™ BCA Protein Assay Kit (Thermo Fisher Scientific; 23225). To separate alpha-synuclein protein, 12% SDS-PAGE (Bio-Rad; Mini-PROTEAN® System) was carried out, and 20 µg protein sample along with protein ladder (PUREGENE Genetix; PG-PMT2922) were loaded into the wells. The protein was then transferred to the activated PVDF membrane (0.2 µm; Parablot; 741260) using semi-dry transfer (Thermo Fisher Scientific; PierceG2 Fast Blotter). Non-specific sites were blocked using 5% bovine serum albumin (MP Biomedicals; BSASG100) solution and were incubated overnight at 4°C with primary antibodies [rabbit monoclonal anti-alpha-synuclein (Abcam; ab138501) and rabbit polyclonal to anti-actin (Abcam; ab1801)] at 1:1000 dilution, followed by washing with 0.1% PBST (0.1% Tween-20 (Sigma-Aldrich; P9416-50ML) in PBS) and incubation with secondary antibody [goat anti-rabbit IgG H&L (HRP) preadsorbed (Abcam; ab7090)] at 1:5000 dilution. Chemiluminescence detection was performed using a chemi-doc (Invitrogen; iBright 1500), and densitometric analysis was performed using ImageJ software.

#### ROS assay

Briefly, the stage-synchronised young-adult worms were harvested in M9 buffer to remove excess adherent bacteria and washed three times with 1× PBS. The worms were stained using DHE (Invitrogen; D1168) (5 µM, in 1× PBS) and incubated for 45 min on a rotor shaker. H_2_O_2_ (Sigma-Aldrich; 323381-500GML) was used as a positive control for each group, and 30 mM ascorbic acid (Sigma-Aldrich; 95209-250G) was used as an antioxidant for the rescue experiment. Post-incubation washing was done using 1× PBS, and a final wash was done using M9 buffer (Gusarov et al., 2021). Worms were immobilized in 100 mM sodium azide (Sigma-Aldrich; 71289) solution for 5 min. Approximately 20 µl of the sample was placed onto glass slides, and images were taken using a Rhodamine filter on a Carl Zeiss Axio Imager.M2 microscope. Fluorescence intensity was quantified using ImageJ software.

#### MMP analysis

For measuring the MMP under *sir-2.2* KD conditions, we first isolated cells using a standard protocol (Zhang et al., 2011). Briefly, young-adult worms of the control and *sir-2.2* gene-silenced condition were washed twice with M9 buffer to remove excess adherent bacteria, re-suspended and washed twice using double-distilled water. Then, 100 μl of the worm pellet was incubated in 200 μl SDS-DTT [0.25% SDS (Sigma-Aldrich; L3771), 200 mM DTT (Thermo Fisher Scientific; R0861), 20 mM HEPES (Sigma-Aldrich; H3375), pH 8.0 and 3% sucrose (HIMEDIA; PCT0607)] solution for 4 min at room temperature; this was followed by the addition of 800 μl egg buffer [118 mM NaCl (HIMEDIA; MB023), 48 mM KCl (HIMEDIA; PCT0012), 2 mM CaCl_2_ (HIMEDIA; GRM710), 2 mM MgCl_2_ (HIMEDIA; GRM728), 25 mM HEPES (Sigma-Aldrich; H3375), pH 7.3] and centrifugation at 12,470 ***g*** for 1 min. Then, the sample were washed five times with egg buffer and centrifuged at 12,470 ***g*** for 1 min each. After this, the worm cuticle was disrupted using 15 mgml^−1^ pronase (from *Streptomyces griseus*; Sigma-Aldrich; 10165921001) enzyme with repeated pipetting for 20 min at room temperature. This was followed by termination of digestion using L-15 medium (Sigma-Aldrich; L1518) and centrifugation at 180 ***g*** for 5 min at 4°C to sediment the cells. Cells were re-suspended in 1 ml L-15 medium and left on ice for 15 min to allow debris to settle. Then, 800 μl supernatant was transferred to a fresh microcentrifuge tube and centrifuged at 180 ***g*** for 5 min at 4°C. After cell isolation, MMP analysis was performed (Shukla et al., 2021 preprint). For this, ∼1×10^5^ cells ml^−1^ were re-suspended in serum-free RPMI (Gibco-Thermo Fisher Scientific). For staining, JC-1 dye (2.5 μgml^−1^; Abcam; ab113850) was used and incubated for 15 min at room temperature. MMP analysis was performed using a flow cytometer with excitation at 488 nm and emission at 530 nm and 590 nm.

## Supplementary Material

10.1242/dmm.052197_sup1 Supplementary information
