# Supplementary material for: Knockdown of mitochondrial sirtuin sir-2.2 reduces alpha-synuclein clearance and impairs energy homeostasis in a model of ageing
Source: Dis Model Mech. 2025 Nov 10;18(11):dmm052197. doi: 10.1242/dmm.052197 (PMC12661644; doi:10.1242/dmm.052197)
Supplement: Supplementary information [file dmm-18-052197-s1.pdf]

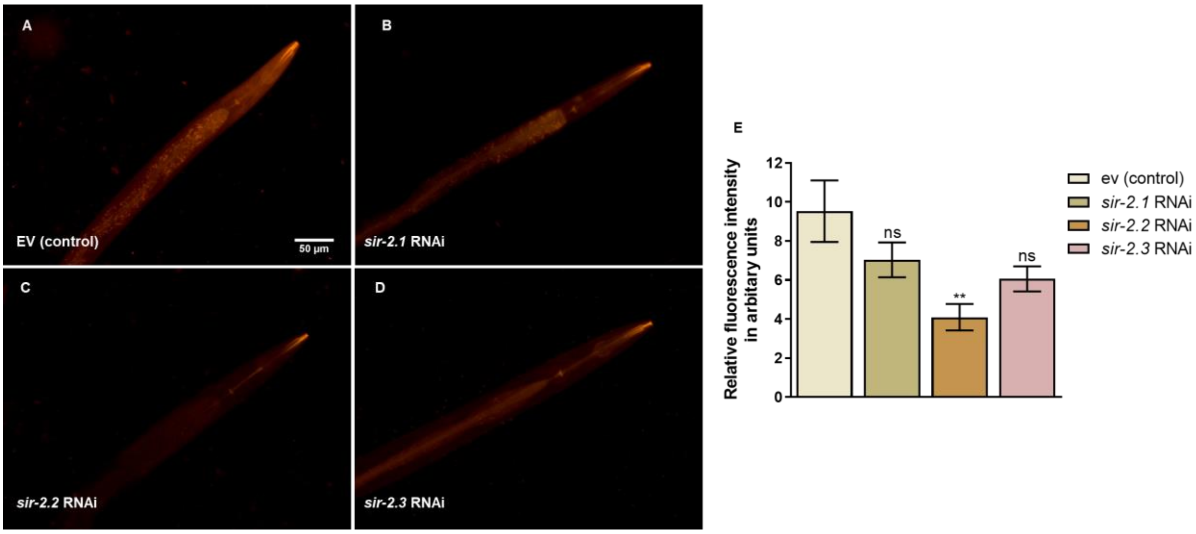

Fig. S1. Initial screening for assessing mitochondrial health using MitoTracker dye in transgenic strain of *C. elegans* (NL5901), under the influence of *sir-2.1, sir-2.2* and *sir-2.3* RNAi. Statistical analysis was done using GraphPad Prism 8 software, employing unpaired t-test (Mean ± SEM; **: $p \leq 0.01$, ns: non-significant). Scale bar: 50 µm.

Disease Models & Mechanisms • Supplementary information

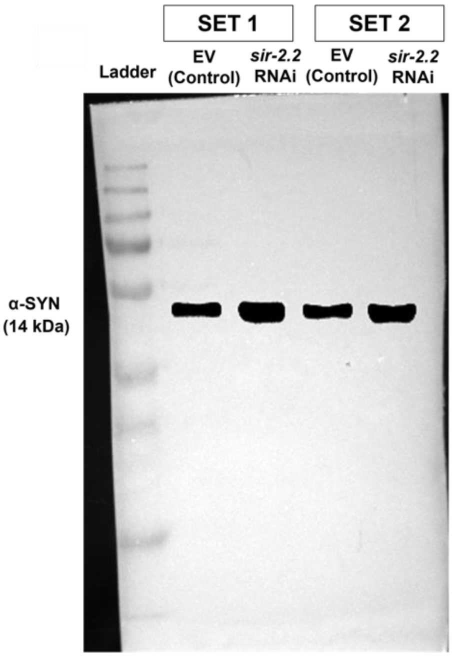

Fig. S2. The full blot image of alpha synuclein protein expression via western blot analysis, showing increased alpha-synuclein aggregation after *sir-2.2* gene RNAi in PD model, that is NL5901 strain of *C. elegans*.

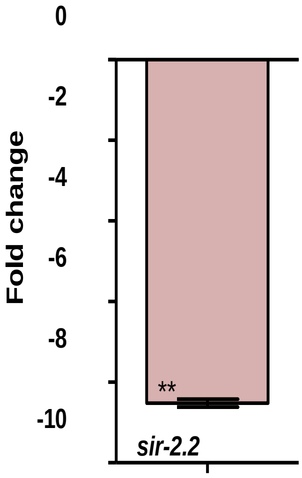

Fig. S3. Graphical representation for validation of *sir-2.2* gene knockdown via RNAi, employing real-time PCR analysis. Statistical analysis was done using GraphPad Prism8 software, employing unpaired t-test (Mean ± SEM; **: p ≤0.01).

Disease Models & Mechanisms • Supplementary information

Table S1. List of primers used in the study.

S. No.	Gene	Forward primer	Reverse primer
1.	*sir-2.2*	GGTATCCCAGATTACCGCTCG	CCAAATCTCGGCCAGGCTAA
2.	*act-1*	TTACTCTTTCACCACCACCGCTGA	TCGTTTCCGACGGTGATGACTTGT
3.	*nsy-1*	TCTTCATTCCACGTTGTGCCATGC	ACCCTCCAGAATTTGCTTCCCGTA
4.	*akt-1*	TCGAGCAAAGCCTAAGGAAGGACA	AACTTCTGCCGACTCCGCATAGAA
5.	*pmk-1*	GTACCGGAGCATATGGAACTG	ATGTACGACGGGCATGAATTA
6.	*sta-2*	CTCCGACGTCGATCTCATTTATC	GATTTCGTCGGGAGCAAGAT
7.	*tir-1*	GGCGAAGAGGTTCCCTATAAAC	CGAACTTCTCAACGTACTCCTC
8.	*sek-1*	GTACGGTTCTATGGAGCAATGT	TTGTATGCGTGCCGGTAAA
9.	*daf-16*	GCGAATCGGTTCCAGCAATTCCAA	ACACGATCCACGGACACTGTTCAA
10.	*skn-1*	TACAGAACGTCCAACCACATC	GCCCTTCTCTCCAGCAATATC
11.	*pink-1*	AGTCGTCTGGACAAAGTGATG	TTGCTCGAAGTTGTCGTTCT
12.	*pdr-1*	CAGACGTCGTACAGCGAATAC	TCATAGGGCTCCCAGAAGAA

Disease Models & Mechanisms • Supplementary information

13.	*mff-1*	AGCGTCGCTCTTCCATTTAC	AGCGTCGCTCTTCCATTTAC
14.	*drp-1*	CTTCGGAGCCTATCAAGTTAGC	GTCCATCGCATCAGTTCCTT
15.	*fzo-1*	TTCCGAAGAACAGGCAATGA	TCTCCAACCAACAGCCTTATAC
16.	*eat-3*	GTCATACAACACCTCGGACAA	CCAGATCCTCTGGGAAAGATTC
17.	*sod-2*	GAGGCGGTCTCCAAAGGAAA	CCAGAGATCCGAAGTCGCTC
18.	*sod-3*	CTCCAAGCACACTCTCCCAG	TCCCTTTCGAAACAGCCTCG
19.	*ctl-2*	CTACAGTCGGTGGTGAGAGC	TACCCATCTGGGAGTCCTCG
20.	*hsp-70*	CAGACGAAGCAGTAGCCTTT	GTGGGACAACATCAACGAGTA
21.	*clpp-1*	GCAGTGTAACAGCAGGACTT	CAGCAGTGATCCCATTGATGA
22.	*atfs-1*	CACATGGTCATCAGCAACAAC	GGGAGCCCATATTATCTCCAAA
23.	*jnk-1*	TGGCTGGTTCCATCATCATCTGGT	CGTTTGAGAACAACCATCTGCGCT
24.	*dat-1*	TTAACCTGCCATGGGCATCCTGTA	TTGCTGTTCCGTCTTCTGACCAGT
25.	*sqst-1*	ACCCGTGCAGCCATTATT	GAATTTGGTCATTCCTGCTTCC
26.	*lgg-1*	CAGAAAGTACCCAGACCGTATT	GTGGTCATGGTTTGTGGAATG
27.	*lgg-2*	TAGCCACGCTTCAAAGTTACA	GTAAACTGCGTTCTCCATCAAATC
28.	*atg-3*	TGCAAACACGCCGAAATG	GCCTGCACGAATTTCAAGAATA
29.	*atg-9*	TGGAAGGGAAAGGCACATAC	ACAGCGAAGCTACACACATC

Disease Models & Mechanisms • Supplementary information

30.	*atg-4.1*	CTGGCGTTTCGATGAAGATTG	CTGCGTCTTCTTCTTCTGTATGA
31.	*bec-1*	CGACOCCAACGAAGCCATATT	GAAGAGCGTCAGAGCAATCA
32.	*atg-18*	AGGAACTATCGCGTGTCTAAAG	GAGCCGATGTCCATTTGGTA

Disease Models & Mechanisms • Supplementary information
